# Urticaria relapse after mRNA COVID‐19 vaccines in patients affected by chronic spontaneous urticaria and treated with antihistamines plus omalizumab: A single‐center experience

**DOI:** 10.1111/dth.15838

**Published:** 2022-09-27

**Authors:** Vincenzo Picone, Maddalena Napolitano, Fabrizio Martora, Luigi Guerriero, Gabriella Fabbrocini, Cataldo Patruno

**Affiliations:** ^1^ Section of Dermatology, Department of Clinical Medicine and Surgery University of Naples Federico II Naples Italy; ^2^ Department of Medicine and Health Sciences Vincenzo Tiberio University of Molise Campobasso Italy; ^3^ Department of Health Sciences University Magna Graecia of Catanzaro Catanzaro Italy

**Keywords:** acute urticaria, chronic spontaneous urticaria, COVID‐19, mRNA vaccines, wheals

## Abstract

Urticaria is a disease characterized by wheals and/or angioedema. Chronic spontaneous urticaria (CSU) occurs for longer than 6 weeks and appears independently of any identifiable exogenous stimulus. During the vaccination campaign for Coronavirus disease 2019 (COVID‐19) pandemic, several cutaneous adverse events have been described, among which urticaria lasting less than 6 weeks (acute urticaria, AU). AU due to vaccines can be IgE or non‐IgE mediated; the former typically develop within 4 h of drug exposure, the latter occurs later and the mechanism is unclear. In this retrospective study we analyzed the frequency and clinical characteristics of urticaria occurring after COVID‐19 vaccine (post‐vaccination urticaria relapse) in adult CSU patients treated with antihistamine and omalizumab, and in clinical remission.

## 1 INTRODUCTION

Urticaria is a disease characterized by wheals and/or angioedema.^1^ Chronic spontaneous urticaria (CSU) occurs for longer than 6 weeks, and appears independently of any identifiable exogenous stimulus.^1^ Type I (IgE antibodies to self‐antigens) or type IIB (mast cell‐directed activating autoantibodies) autoimmunity are the mechanisms leading to mastocyte degranulation in most cases of CSU.^2^ The first‐line therapy of CSU is second‐generation H1 antihistamines; omalizumab, a humanized recombinant monoclonal anti‐IgE antibody, can be added to antihistamines in refractory patients.^1^

During the vaccination campaign for Coronavirus disease 2019 (COVID‐19) pandemic, several cutaneous adverse events have been described, among which urticaria lasting less than 6 weeks (acute urticaria, AU).^3^, ^4^ AU due to vaccines can be IgE or non‐IgE mediated; the former typically develop within 4 h of drug exposure, the latter occurs later and the mechanism is unclear.^3^, ^4^, ^5^

## 2 MATERIAL AND METHODS

**FIGURE 1:**
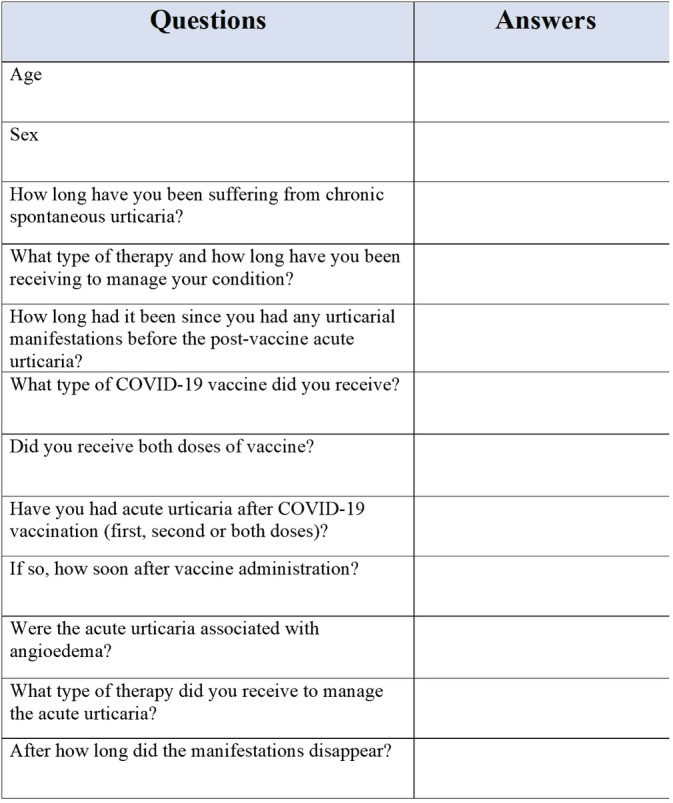
Anonymous questionnaire administered to each CSU patient who developed urticaria after COVID‐19 vaccine

In this retrospective study we analyzed the frequency and clinical characteristics of urticaria occurring after COVID‐19 vaccine (post‐vaccination urticaria relapse, PVUR) in adult CSU patients treated with antihistamine and omalizumab, and in clinical remission. For this purpose, an interview was conducted at the Dermatology Unit of the University Federico II of Naples using an anonymous questionnaire (Figure 1). Inclusion criteria were: age ≥ 18 years, diagnosis of CSU, continuous therapy for at least 3 months with second‐generation H1 antihistamines plus omalizumab, and weekly Urticaria Activity Score (UAS7) of 0 for at least 4 weeks before vaccine administration. The following parameters were analyzed for each patient: sex, age, previous adverse drug reactions, drugs other than antihistamine and omalizumab, time of CSU onset, period (weeks) of CSU remission, and type of COVID‐19 vaccine received. In addition, data regarding the skin rash were recorded in patients reporting PVUR: occurrence after first/second/both vaccine doses, latency after vaccination, association with angioedema, duration, and treatment. Descriptive statistics were calculated for each demographic and clinical variable, using frequencies and percentage for categorical variables, and mean ± SD for continuous variables.

## 3 RESULTS

Sixty (25 males; mean age: 36 ± 12.53 years) patients were enrolled. The mean CSU duration was of 24 ± 8.86 months. Fifty (83.3%) had received COVID‐19 vaccine. In all the cases, messenger RNA (mRNA) vaccine [45 (75.0%) Comirnaty® (Pfizer® BNT162b2) and 5 (8.3%) Spikevax® (Moderna® mRNA‐1273)] had been administered. All the patients were under treatment with second‐generation H1 antihistamine plus omalizumab, with a mean treatment duration of 9 ± 3.35 months. CSU was currently in clinical remission (UAS7 = 0) in all the patients, on average from 8 ± 4.12 weeks. Four (8.0%) patients reported PVUR documented by a physician (emergency room or general practitioner). In 3 (6.0%) cases PVUR occurred after Comirnaty® and in 1 (2.0%) case after Spikevax® (Table 1). One (2.0%) patient developed the rash after the first dose of Comirnaty®, while 3 (6.0%) after the second dose [2 Comirnaty® and 1 Spikevax®]. Two (4.0%) of them showed PVUR after a few minutes following the second dose of Comirnaty®. Only 1 (2.0%) showed also angioedema. One (2.0%) patient developed the flare about 24 h after the first dose of Comirnaty®, not relapsing after the second dose of the same vaccine. Angioedema was reported by 1 (2.0%) patient about 18 h following the second dose of Spikevax®. No patient had urticaria after both doses. A short course of systemic corticosteroid led to flare healing in all the patients in 18 ± 5.15 h.

**TABLE 1:** Demographics and clinical characteristics of CSU patients who developed urticaria after COVID‐19 vaccine

	Patient 1	Patient 2	Patient 3	Patient 4
Age/sex	21/F	31/F	44/M	50/M
Duration of CSU (months)	24	24	36	60
Comorbidity	Asthma		Autoimmune thyroiditis	
Ongoing CSU therapy	Cetirizine + omalizumab	Cetirizine + omalizumab	Cetirizine + omalizumab	Cetirizine + omalizumab
COVID‐19 vaccine	Comirnaty® (BNT162b2)	Comirnaty® (BNT162b2)	Comirnaty® (BNT162b2)	Spikevax® (mRNA‐1273)
Dose of vaccine followed by PVU	2°	2°	1°	2°
Time of CSU remission before PVU (weeks)	4	4	16	8
Latency of PVU from vaccination	5 min	10 min	24 h	18 h
Associate angioedema	Yes	No	No	Yes
Duration of PVU (hours)	24	20	12	16
Adjunctive therapy for PVU	Prednisone 25 mg/die	Prednisone 25 mg/die	Prednisone 25 mg/die	Prednisone 25 mg/die

Abbreviations: CSU, chronic spontaneous urticaria; PVU, post‐vaccination urticaria.

## 4 DISCUSSION

Currently, there are few data on the occurrence of urticaria flare after COVID‐19 vaccines in CSU treated patients.^6^ Anecdotal cases of CSU induced by COVID‐19 vaccine are described.^7^ In our experience, 8.0% of CSU patients under successful treatment with antihistamine and omalizumab reported urticaria after mRNA vaccine. In two cases the flare occurred few minutes after the vaccine administration, whereas in other two subjects several hours later (>4 h). In all the patients, the episode healed with a short course of systemic corticosteroid. Both immediate (<4 h) and late AU have been described in general population after mRNA COVID‐19 vaccine.^3^, ^4^, ^5^ In immediate AU, type I hypersensitivity mediated by IgE antibodies to a drug component has been proposed.^8^ To date, the main culprits in the induction of IgE mediated, immediate reactions to COVID‐19 vaccine is polyethylene glycol (PEG)‐200 contained in both mRNA vaccines, tromethamine contained in Spikevax®, or polysorbate‐80 contained in vector‐based (Adenovirus) vaccine Vaxzevria (Astra‐Zeneca ChAdOx1‐S).^9^ The cause of late AU is instead unclear and could be associated with the action of viral‐like spike protein induced by vaccine, mimicking urticaria sometimes associated with COVID‐19.^10^, ^11^ In an observational study on a cohort of 2740 Italian individuals selected from the general population, AU was found in 14 cases (0.5%).^4^ In our experience, the percentage of CSU patients experiencing PVUR was significantly higher (8.0%). This might be influenced by the main limitations of this study consisting in the small number of patients (*n* = 50), its retrospective, questionnaire‐based nature, and the diagnosis made elsewhere by a non‐specialist physician. Some authors hypothesize that in patients with CSU there could be a greater predisposition to develop urticaria flare after environmental stimuli, such as COVID‐19 vaccines.^12^ This observation could make the mechanisms proposed for both immediate and late AU due to COVID‐19 vaccine in general population different from urticaria flare occurring in patients with CSU. Our experience might support this hypothesis. Furthermore, none of our patients had been treated with Adenovirus vaccine. Some other authors described urticaria in CSU patients treated with omalizumab only after mRNA vaccine and not after Adenovirus vaccine.^13^

The literature suggests that, in case of AU after COVID‐19 vaccine, a change of vaccine type is recommended for the next dose^12^; however, one of our patients who experienced urticaria flare after the first dose of Comirnaty® safely received the second dose of the same vaccine. It has been reported that most patients with immediate AU to mRNA COVID‐19 vaccine, especially to the first dose, are likely previously sensitized to PEG or tromethamine contained in some drugs or preparation for medical exams or surgery.^14^ Of note, none of our four patients with urticaria after COVID‐19 vaccine reported having been exposed to these products in the past. At the moment of vaccination, all our patients were on therapy with antihistamine and omalizumab, a monoclonal antibody whose efficacy in the therapeutic management of CSU has been widely demonstrated.^1^ Notwithstanding, 8% of patients under this treatment showed PVUR compared to 0.5% of general population.^4^ Therefore, it seems that these drugs are efficacious in controlling CSU but not in preventing vaccine‐related urticaria in these patients. Indeed, a short course of systemic corticosteroid was prescribed to all the patients, with healing of the rash. Corticosteroids could interfere with immune response to vaccine; therefore, higher dosage of antihistamines should be preferable for both the treatment and the prevention of vaccine induced urticaria flare in CSU patients.^12^ On the other hand, omalizumab has been reported to be efficacious in preventing anaphylactoid reactions to mRNA COVID‐19 vaccine in patients not affected with CSU.^15^ The pathogenesis of urticaria flare in CSU patients after COVID‐19 vaccine is doubtful. In our opinion, PVUR should be considered as a relapse of CSU and not as AU related to the vaccine. More data on larger populations are needed to draw stronger conclusions. This is also to give a correct information on COVID‐19 vaccine to CSU patients that are not infrequently perplexed by vaccination.

## CONFLICT OF INTEREST

Cataldo Patruno acted as investigator, speaker, consultant, and/or advisory board member for AbbVie, Amgen, Eli Lilly, Leo Pharma, Novartis, Pfizer, Pierre Fabre, and Sanofi. M. Napolitano acted as speaker, consultant and/or advisory board member for Abbvie, Eli Lilly, Leo Pharma, Novartis, and Sanofi. G. Fabbrocini acted as a speaker or consultant for Abbvie, Amgen, Eli Lilly, Janssen, Leo‐Pharma, Almyrall, Novartis, and UCB. None of the contributing authors has any conflict of interest, including specific financial interests of relationships and affiliation relevant to the subject matter or discussed materials in the manuscript.

## INFORMED CONSENT

The patients in this manuscript have given written informed consent to the publication of their case details.

## Data Availability

Data sharing is not applicable to this article as no new data were created or analyzed in this study.
